# Disruption of carotene biosynthesis leads to abnormal plastids and variegated leaves in *Brassica napus*

**DOI:** 10.1007/s00438-020-01674-w

**Published:** 2020-04-18

**Authors:** Xiaobin Zhao, Kaining Hu, Mengjiao Yan, Bin Yi, Jing Wen, Chaozhi Ma, Jinxiong Shen, Tingdong Fu, Jinxing Tu

**Affiliations:** National Key Laboratory of Crop Genetic Improvement, National Sub-Center of Rapeseed Improvement in Wuhan, Huazhong Agricultural University, Wuhan, People’s Republic of China

**Keywords:** *Brassica napus*, Variegation, Chloroplast, Carotene biosynthesis, Reactive oxygen species

## Abstract

Leaf color is an important characteristic of normal chloroplast development. Variegated plants have green- and white-sectored leaves, which can be used to identify important pathways and molecular mechanisms of chloroplast development. We studied two *Brassica napus* variegation mutants from same one variegated ancestor, designated ZY-4 and ZY-8, which have different degrees of variegation. When grown in identical conditions, the ratio of white sectors in ZY-4 leaves is higher than in ZY-8. In both mutants, the cells in green sectors contain normal chloroplasts; while, the cells in white sectors contain abnormal plastids. Seedling chloroplasts ultrastructure of both mutants showed that the biogenesis of chloroplasts was blocked in early stages; delayed development and structual damage in ZY-4 were more serious than in ZY-8. Employing bulked segregant analysis(BSA), two bulks (BY142 and BY137) from BC_2_F_1_ lines derived from ZY-4 and ZS11, and one bulk (BY56) from BC_2_F_1_ lines derived from ZY-8 and ZS11, and screening by Brassica 60K SNP BeadChip Array, showed the candidate regions localized in chromosome A08 (BY142), C04 (BY137), and A08 (BY56), respectively. Transcriptome analysis of five seedling development stages of ZY-4, ZY-8, and ZS11 showed that photosynthesis, energy metabolism-related pathways and translation-related pathways were important for chloroplast biogenesis. The number of down- or up-regulated genes related to immune system process in ZY-4 was more than in ZY-8. The retrograde signaling pathway was mis-regulated in both mutants. DEG analysis indicated that both mutants showed photooxidative damages. By coupling transcriptome and BSA CHIP analyses, some candidate genes were identified. The gene expression pattern of carotene biosynthesis pathway was disrupted in both mutants. However, histochemical analysis of ROS revealed that there was no excessive accumulation of ROS in ZY-4 and ZY-8. Taken together, our data indicate that the disruption of carotene biosynthetic pathways leads to the variegation phenotypes of ZY-4 and ZY-8 and there are some functions that can compensate for the disruption of carotene biosynthesis in ZY-4 and ZY-8 to reduce ROS and prevent seedling mortality.

**Electronic supplementary material:**

The online version of this article (10.1007/s00438-020-01674-w) contains supplementary material, which is available to authorized users.

## Introduction

Chloroplast are the most important organelles in plant cells. Chloroplasts host numerous essential metabolic pathways including photosynthesis, the manufacture of fatty acids and pigments, and the synthesis of amino acids (Gutierrez-Nava Mde et al. 2004; Zoschke and Bock 2018). As semi-autonomous organelles, the proteome of today’s chloroplasts consists of ~ 3000 proteins, most of which are nucleus encoded and post-translationally imported into the chloroplast (Zoschke and Bock 2018). Chloroplast development in plants is regulated by coordinated expression of nuclear and chloroplast genomes plus a series of coordinated biological processes. Obvious activities include RNA processing, the import of nuclear-encoded proteins, protein maturation and degradation, and the establishment of a thylakoid network (Waters and Langdale 2009). Chloroplast differentiation commences from proplastids, which are simple, undeveloped progenitors that are present in shoot meristematic tissues and root apex. They are non-photosynthetic, small, round-shaped organelles that contain few internal membranes mostly as vesicles or small saccular structures. The maturation process is light induced and starts with the formation of a long lamellae inside a proplastid, resulting in the formation of juvenile chloroplasts characterized by lens-shaped flattened appearances with parallel layered grana, but smaller than mature chloroplasts. Later, these lamellae are transformed into disc-shaped structures that assemble into grana stacks. Eventually, the complex interconnected thylakoid membrane network arises in mature chloroplasts (Mechela et al. 2019).

Many mutants defective in chloroplast development have been identified and classified into four classes based on the severity of the phenotype on plant growth and development: embryo lethal, seedling lethal or albino, seedling lethal but conditional survival, and autotrophy with pale-green, virescent, or variegated leaves (Kim et al. 2009). Variegated plants typically have green- and white-sectored leaves, providing a means of studying the expression of genes important for chloroplast development. However, mutant analysis is difficult, either because mutations in a gene of interest are lethal or because they do not show a readily distinguishable phenotype. Cells in the green sectors contain normal-appearing chloroplasts; whereas, cells in the white sectors lack pigments and appear to be blocked at various stages of chloroplast biogenesis (Yu et al. 2007).

Extensive studies have been conducted on the molecular mechanism of leaf variegation. A multitude of variegation mutants have been isolated by genetic approaches, and most of these genes are in the nucleus and encode proteins used within chloroplasts.

In the Arabidopsis white–green variegated mutant *immutans* (*im*), the lack of plastid terminal oxidase IM results in the production of reactive oxygen and, therefore, causes the formation of photo-oxidized plastids in the white sectors of *im* (Carol et al. 1999; Wu et al. 1999). The green sectors contain cells with morphologically normal chloroplasts; whereas, the white sectors contain vacuolated plastids that lack organized lamellar structures (Wetzel et al. 1994; Aluru et al. 2001). The albino sectors of *im* plants contain reduced levels of carotenoids and increased levels of the carotenoid precursor phytoene (Wetzel et al. 1994; Norris et al. 1995; Carol et al. 1999). The function of IM appears to be essential to prevent photooxidative damage during early steps of chloroplast formation (Carol et al. 1999; Wu et al. 1999; Aluru et al. 2009; Rosso et al. 2009). Oxidative stress responses are largely induced in *im* white tissues; however, *im* green sectors develop additional energy-dissipating mechanisms that can allow for the formation of green sector (Wetzel et al. 1994; Aluru et al. 2009).

FtsH proteins belong to the AAA (ATPase associated with various cellular activities) ATPase superfamily, and are anchored by a single transmembrane domain in thylakoids (Yu et al. 2007). Thylakoid FtsH complexes comprise four members of FtsH proteins, FtsH1 and FtsH5 (type A), and FtsH2 and FtsH8 (type B), in which VAR2/AtFtsH2 is one of the most abundant subunits. Biochemical analysis suggested that thylakoid FtsHs are required to degrade the photodamaged reaction center protein D1 during the Photosystem II repair cycle (Liu et al. 2019). All normal green tissues and organs of *var2*, except for cotyledons, are variegated, and the green sectors arise from white sectors during early leaf expansion, and sector formation is irreversible in the mature leaves (Putarjunan et al. 2013). Multiple genetic screens for *var2* suppressors in several laboratories have revealed that many suppressors of *var2* are genetic factors involved in chloroplast transcription, translation and post-translational turnover. Comprehensive genetic analyses with *var2* suppressors defective in chloroplast translation have established that the enhancement of *var2* leaf variegation by cytosolic ribosomal protein mutants depends upon chloroplast translation (Wang et al. 2018).

The Arabidopsis Thylakoid formation1 (*Thf1*) gene encodes an important chloroplast protein, which controls a step required for the organization of vesicles into mature thylakoid stacks. Green sectors of *Thf1*-disrupted leaves contain some chloroplasts that form organized thylakoid membranes, indicating that an inefficient compensatory mechanism supports thylakoid formation in the absent of *Thf1* (Wang et al. 2004). PRPS9 and PSRP5 are plastid ribosomal proteins that are second-site mutations and suppress leaf variegation of *thf1-1*. SIG6 is a plastid transcription factor specifically controlling gene expression through the plastid-encoded RNA polymerase and the mutation of SIG6 suppresses *thf1* variegation (Hu et al. 2015). Genetic screening for the second-site suppressor lines of *thf*1 and *var2* suggested that the reduced rate of plastid protein biosynthesis is important for chloroplast development in variegated leaves (Ma et al. 2015b).

Although those studies of mutants improved our understanding of variegation mechanism in Arabidopsis, research on the *Brassica napus* variegated mutant is lacking, only four chlorophyll-deficient mutants had been reported. The yellow–green leaf phenotype in *Cr3529* (Zhao et al. 2000; Wang et al. 2008), the chlorophyll-deficient mutant *Bnchd1* (Zhao et al. 2013), the chlorophyll-deficient mutant *BnC.ygl* (Zhu et al. 2014, 2017), and the chlorophyll-deficient mutant *cde1* (Wang et al. 2016).

In this study, we characterized two variegated mutants derived from a same one ancestor, designated ZY-4 and ZY-8. The BSA and Brassica 60K SNP BeadChip Array was used to locate the candidate regions. To gain further insight into the molecular mechanisms of two mutants, we compared the transcriptomes of ZY-4 and ZY-8 to ZS11, and analyzed the DEGs of each comparison. Coupling transcriptome and BSA CHIP analyses results, candidate genes and important pathways were identified.

## Materials and methods

### Plant materials, population construction and growth conditions

ZY-4 and ZY-8 were from a same variegated mutant that was discovered at the experimental plot of Huazhong Agricultural University, Wuhan, China. This variegated mutant was open pollinated to produce a F_1_ generation. All F_1_ plants were normal green. The F_1_ plants were self-pollinated to produce F_2_ population. There were 10 variegated plants in F_2_ population, designated ZY-1–ZY-10. Ten variegated plants were self-pollinated to produce generations. Plants phenotype of generations of ZY-4 and ZY-8 were stable ever at Wuhan and Lanzhou, China.

Conventional cultivar ‘ZS11’ was provided by Department of Rapeseed Research at Huazhong Agricultural University. ‘ZY-4’ and ‘ZY-8’ were crossed with ‘ZS11’ to produce F_1_ generations. The F_1_ plants were backcrossed with ‘ZY-4’ or ‘ZY-8’, respectively, to produce BC_1_F_1_ lines. Green plants selected randomly from BC_1_F_1_ were crossed with variegated plants selected randomly from BC_1_F_1_ to produce BC_2_F_1_ populations. All BC_2_F_1_ subpopulations were assayed for their segregating ratio. We successfully constructed two BC_2_F_1_ subpopulations form ZS11 × ZY-4 (designated BY137, BY142) and one BC_2_F_1_ subpopulation form ZS11 × ZY-8 (designated BY56), with a 1:1 segregation.

The plants for the seeding morphology analysis, BSA and Brassica 60K SNP BeadChip Array analysis were grown in the experimental plot of Huazhong Agricultural University, and the plants for the young seeding morphology analysis, RNA-seq and histochemical analysis of ROS were grown in soil under 16-h fluorescent white light photoperiods at 23 °C.

### Transmission electron microscopy analysis

Apical meristem and cut leaves (2 mm) were prefixed and lyophilized in 2.5% (w/v) glutaraldehyde with 0.1-M phosphate buffer (pH 7.4), and fixed again in 1% OsO_4_ with the same buffer. The procedures were conducted as previously described (Yi et al. 2010).

### BSA and Brassica 60K SNP beadchip array analyses

BSA combined with the Brassica 60K SNP BeadChip Array was used to identify single-nucleotide polymorphism (SNPs).Total DNA was extracted from fresh leaves using the cetyl-trimethylammonium bromide (CTAB) method (Murray and Thompson 1980). Three bulked-variegated pools and three bulked-normal pools were constructed. Each bulk comprised equal amounts of DNA (200 ng/μl) collected from three individuals which were selected from the BC_2_F_1_ population. The two parents and six DNA bulks for one subpopulation were genotyped using the Brassica 60K SNP BeadChip Array developed by Emei Tongde Co. (Beijing) according to the manufacturer’s protocol (https://www.illumina.com/science/technology/microarray.html). There were three parents (ZY-4, ZY-8, ZS11) and 18 DNA bulks (each six for BY137, BY142, BY56) in this experiment.

Genotyping was performed using GenomeStudio genotyping software (v2011.1; Illumina, Inc.). Polymorphism analysis was performed using the IF function of Excel. The categories used for filtering SNPs linked to the variegated phenotype were as follows: SNPs were (I) polymorphic between two parents; (II) monomorphic between three bulked-variegated pools; (III) monomorphic between three bulked-normal pools; and (IV) polymorphic between bulked-normal and bulked-variegated pools. SNPs between samples from normal and variegated plants were subjected to BLAT searches against the *B. napus* genome (https://www.genoscope.cns.fr/brassicanapus) to determine chromosome positions (*E* value ≤ 2e − 18).

### RNA extraction and RNA sequencing

Cotyledons of seven days after seeded (7 DAS), shoot meristem of 7 DAS, first true leaf of 9 DAS, first true leaf of 11 DAS, and first true leaf of 13 DAS were detached from the seedlings of each mutant and ZS11, immediately frozen in liquid nitrogen, and stored at − 80 ℃ for total RNA isolation. There were three repeated samples at each stage for ZY-4, ZY-8 and ZS11. Total RNA was extracted according to the TRIzol method (Chomczynski 1993).

After total RNA extraction and DNaseιtreatment, magnetic beads with Oligo (dT) were used to isolate mRNA. Within the fragmentation buffer, the mRNA was in short fragments. cDNA was synthesized using the mRNA fragments as templates. Short fragments were purified and resolved in EB buffer for end reparation and single nucleotide A (adenine) addition. Afterwards, the short fragments were ligated with adapters. Suitable fragments were selected for the PCR amplification as templates. During the QC (quality control) steps, Agilent 2100 Bioanaylzer and ABI StepOnePlus Real-Time PCR System were used in quantification and qualification of the sample library. All 45 libraries were finally sequenced using Illumina HiSeq™ xten. The raw sequence data were deposited in the NCBI Sequence Read Archive (Accession No. PRJNA559661).

### Data processing and read mapping

After sequencing, the raw reads were filtered. Data filtering included removing adaptor sequences, contaminated or low-quality reads from raw reads. Next, the clean reads from each library were aligned against the *Brassica napus* cultivar ‘ZS11’ genome (Sun et al. 2017) using HISAT2 (Kim et al. 2015) within default settings. Only uniquely mapped reads were considered for further analyses.

### RNA-seq analysis

Based on the gene expression level calculated by feature counts (Liao et al. 2014), ∼59,613–64,505 genes were expressed in the five stages. Statistical analyses were performed using DESeq2 package (Love et al. 2014) in R statistical environment. Based on rlog-transformed counts calculated by the rlog function in the DESeq2 package, a Pearson correlation coefficients heatmap between each pair of biological replicates at each stage were created. We enriched the DEGs (different expression genes), and classified all the functional DEGs and unigenes based on the gene ontology (GO) database through TBtools (Chen et al. 2018).

### Histochemical analysis of ROS

To detect the presence of reactive oxygen species (ROS), nitroblue tetrazolium (NBT) was used as a substrate that forms insoluble diformazan upon reduction. The method (Jabs et al. 1996) was adopted from Jabs T et al. with some modification. A leaf was photographed before it was removed from a plant and quickly placed in 6-mM NBT (in 10-mM Na-Citrate buffer, pH6) until the leaf was fully submerged. The leaf was kept in the dark for two hours at 25 °C to allow the formation of insoluble dark blue diformazan. The treated leaf was immersed in boiling 99% ethanol for 30 min for the removal of pigments except for the diformazan.

## Results

### Seedling morphology analysis and ultrastructure of chloroplasts

**Fig. 1:**
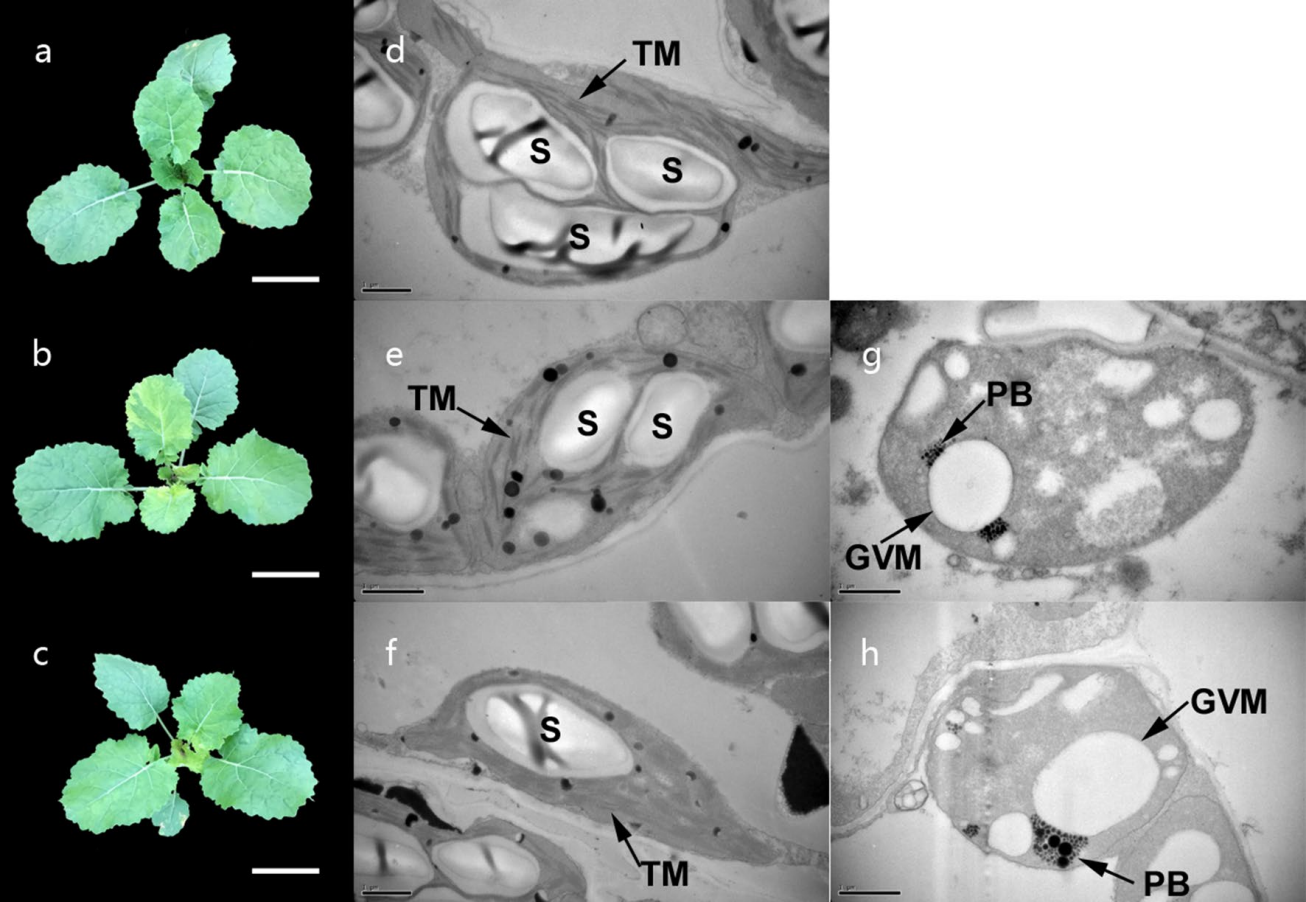
The leaf morphology of ZS11 (**a**), variegation mutant ZY-4 (**b**) and variegation mutant ZY-8 (**c**). New leaves of both mutants were variegated, and the old leaves turned green. Scale bar: 5 cm (**a-c**). Ultrastructure of chloroplasts from ZS11 (**d**), green sectors of ZY-4 (**e**), green sectors of ZY-8 (**f**), green sectors of two mutants had normal chloropalsts; ultrastructure of chloroplasts from white sectors of ZY-4 (**g**), white sector of ZY-8 (**h**), white sectors of two mutants had abnormal chloroplasts. *TM* thylakoid membrane, *S* starches, *PB* plastoglobule, *GVM* globular vacuolated membrane. Scale bar 1 μm (**d–h**)

The *Brassica napus* leaf variegated mutants, ZY-4 and ZY-8, were seeded in experiment plot. When compared with the normal leaves of ZS11, the new leaves of both mutants were variegated, and the old leaves turned green. The ratio of write section in ZY-4 leaf was higher than in ZY-8 (Fig. 1a–c). We analyzed the ultrastructure of chloroplasts and found that, in both mutants, the cells in the green sectors contained normal chloroplasts; while, the cells in the white sectors contained abnormal plastids (Fig. 1d–h). The structure of abnormal plastids in ZY-4 and ZY-8 was similar to each other. These plastids contained the globular vacuolated membrane structure.

### BSA and Brassica 60K SNP beadchip array analyses

We successfully constructed two BC_2_F_1_ lines form ZS11 × ZY-4 (designated BY137, BY142) and one BC_2_F_1_ line form ZS11 × ZY-8 (designated BY56), with a 1:1 segregation at the variegated locus. Of the 52,157 SNPs on the SNP array, there were 23,918 SNPs that were polymorphic between ZS11 and ZY-4. In BY142, 211 SNPs were polymorphic between “variegated” bulks and “normal” bulks (Table S1-1), of which 173 located on chromosome A08, and 166 were localized to a 10- to 15-Mb region of chromosome A08. In BY137, there were 329 SNPs that were polymorphic between different bulks (Table S1-2). Among these SNPs, 275 located on chromosome C04, of which 109 were localized to a 36–37-Mb region of chromosome C04. There were 25,159 that were polymorphic between ZS11 and ZY-8. Of these SNPs, 138 were polymorphic between “variegated” bulks and “normal” bulks of BY56 (Table S1-3). There were 102 SNPs located on chromosome A08, of which 82 were localized to a 11–12-Mb region of chromosome A08.

### Young seedling morphology and ultrastructure of chloroplasts in ZS11, ZY-4 and ZY-8

**Fig. 2:**
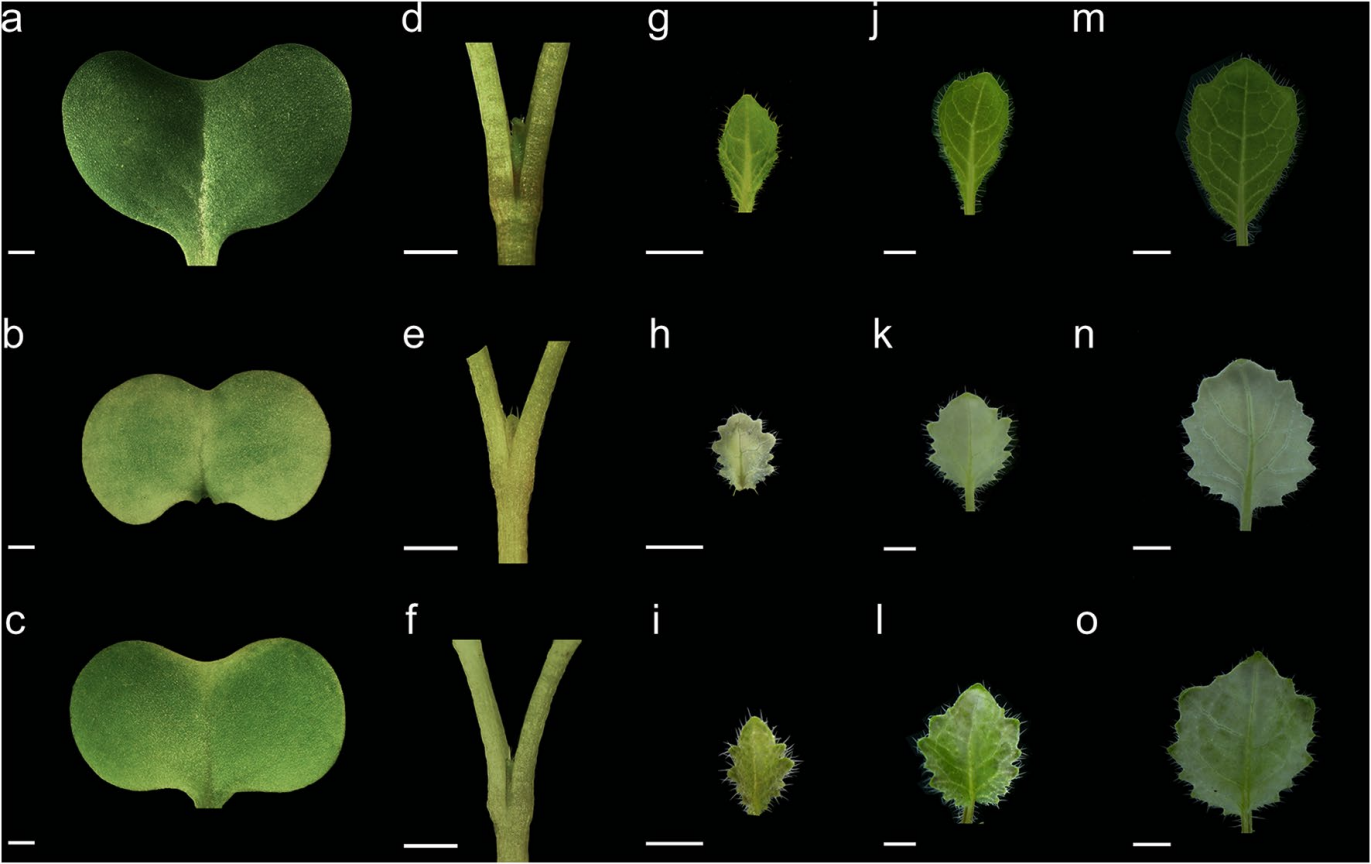
The leaf morphology of five seedling development stages of ZS11(**a**, **d**, **g**, **j**, **m**), ZY-4 (**b**, **e**, **h**, **k**, **n**) and ZY-8 (**c**, **f**, **i**, **l**, **o**). Cotyledon of 7DAS (**a**–**c**), top shoot of 7DAS (**d**–**f**), first true leaf of 9DAS (**g**–**i**), first true leaf of 11DAS (**j**–**l**), first true leaf of 13DAS (**m**–**o**). Cotyledons and ture leaves of two mutants were variegated, and the ratio of white sections in ZY-4 leaves was higher than in ZY-8. DAS, days after seeded. Scale bar 2 mm

To explore the mechanism of variegation, each mutant and ZS11 were planted in soil under 16-h fluorescent white light photoperiods at 23 °C. Changes in morphology were determined seven days after seeded (DAS), 9 DAS, 11 DAS and 13 DAS. No obvious changes were visible in 7 DAS meristem between the mutants and ZS11. While the mutants true leaves at 9 DAS were variegated, and the extent of white part changed with seedling development, the ratio of white sections in ZY-4 leaves was higher than in ZY-8 (Fig. 2).

**Fig. 3:**
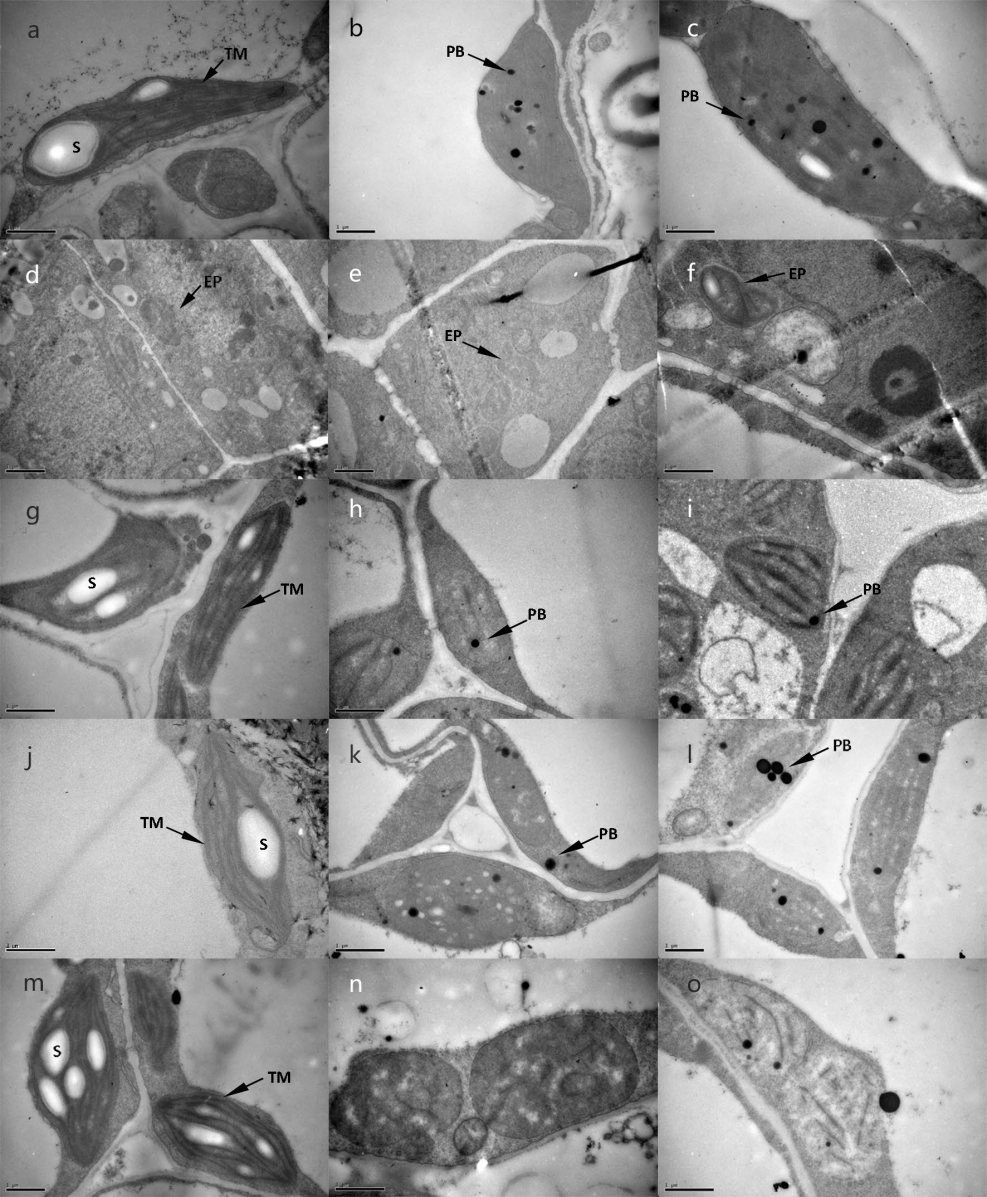
Transmission electron microscopy analysis of five seedling development stages of ZS11 (**a**, **d**, **g**, **j**, **m**), ZY-4 (**b**, **e**, **h**, **k**, **n**) and ZY-8 (**c**, **f**, **i**, **l**, **o**). Cotyledon of 7 DAS (**a**–**c**): chloroplasts of two mutants were almost mature but having an increased number and size of plastoglobules; top shoot of 7 DAS (**d**–**f**): ZY-4 and ZY-8 showed similar morphologies to ZS11; first true leaf of 9 DAS (**g**–**i**): the stage of chloroplasts development in both mutants were pre-chloroplast; first true leaf of 11 DAS (**j**–**l**): two mutants displayed signs of damage to the chloroplast ultrastructure; first true leaf of 13 DAS (**m**–**o**): the ZY-8 lamellar structure was further degradated, and the chloroplasts of ZY-4 were not observed with normal grana stacks. *TM* thylakoid membrane, *PB* plastoglobule, *EP* etioplasts, *S* starches, *DAS* days after seeded. Scale bar: 1 μm

Such leaf variegations of ZY-4 and ZY-8 suggested alterations in chloroplast morphology. To test this possibility, chloroplast structure during the seedling development in both mutants and ZS11 were analyzed by transmission electron microscopy (Fig. 3). At 7 DAS, ZS11 cotyledon chloroplasts were mature, but the cotyledon chloroplasts of two mutants were almost mature but with the remarkable characteristic of an increased number and size of plastoglobules, which are indicative of oxidative stress and senescence (Austin et al. 2006). The chloroplasts of ZS11 developed large starch grains; however, the chloroplasts of ZY-4 and ZY-8 did not develop any starches. The etioplasts in 7 DAS top meristem of ZS11 lacked thylakoid membranes. No obvious differences about the chloroplast ultrastructures were detected between ZS11 and mutant plants at 7 DAS. The first true leaves of ZS11 at 9 DAS had chloroplasts developed almost mature and contained starches. While the chloroplast biogenesis was delayed in both mutants, with ZY-4 delayed more than ZY-8, only a few of stroma lamellae were detected in ZY-4. The stage of chloroplast development in both mutants was pre-chloroplast at 9 DAS. AT 11 DAS, more plastoglobules were found and no starch was accumulated in both mutants. Two mutants displayed signs of damage to the chloroplast ultrastructure. ZY-8 displayed fewer stroma lamellae than at 9 DAS. However, stacked, stromal thylakoid membranes were not observed in ZY-4. At 13 DAS, further degradation of lamellar structure was detected from fragments of stroma lamellae in ZY-8. The chloroplasts of ZY-4 were not observed with normal grana stacks and had membrane structures of varying sizes.

### RNA-seq and DEGs analysis of ZY-4 and ZY-8

To characterize the transcriptional hallmarks of ZY-4 and ZY-8 during seedling development, we analyzed RNA expression profiles extracted from five seedling developmental stages(7 DAS cotyledon, 7 DAS shoot meristem, 9 DAS true leaf, 11 DAS true leaf, 13 DAS true leaf). There were 49,045 and 49,288 common genes that were expressed at each stage in ZY-4 and ZS11, and ZY-8 and ZS11, respectively. A Pearson correlation coefficients heatmap between each pair of biological replicates at each stage were created (Fig. S1). Principal component analysis (PCA) showed that ZS11 and ZY-4, ZS11 and ZY-8 could be clearly identified on the first principal components axis (Fig. S2). These results showed that the sequencing data used in the present study were highly reliable.

**Fig. 4:**
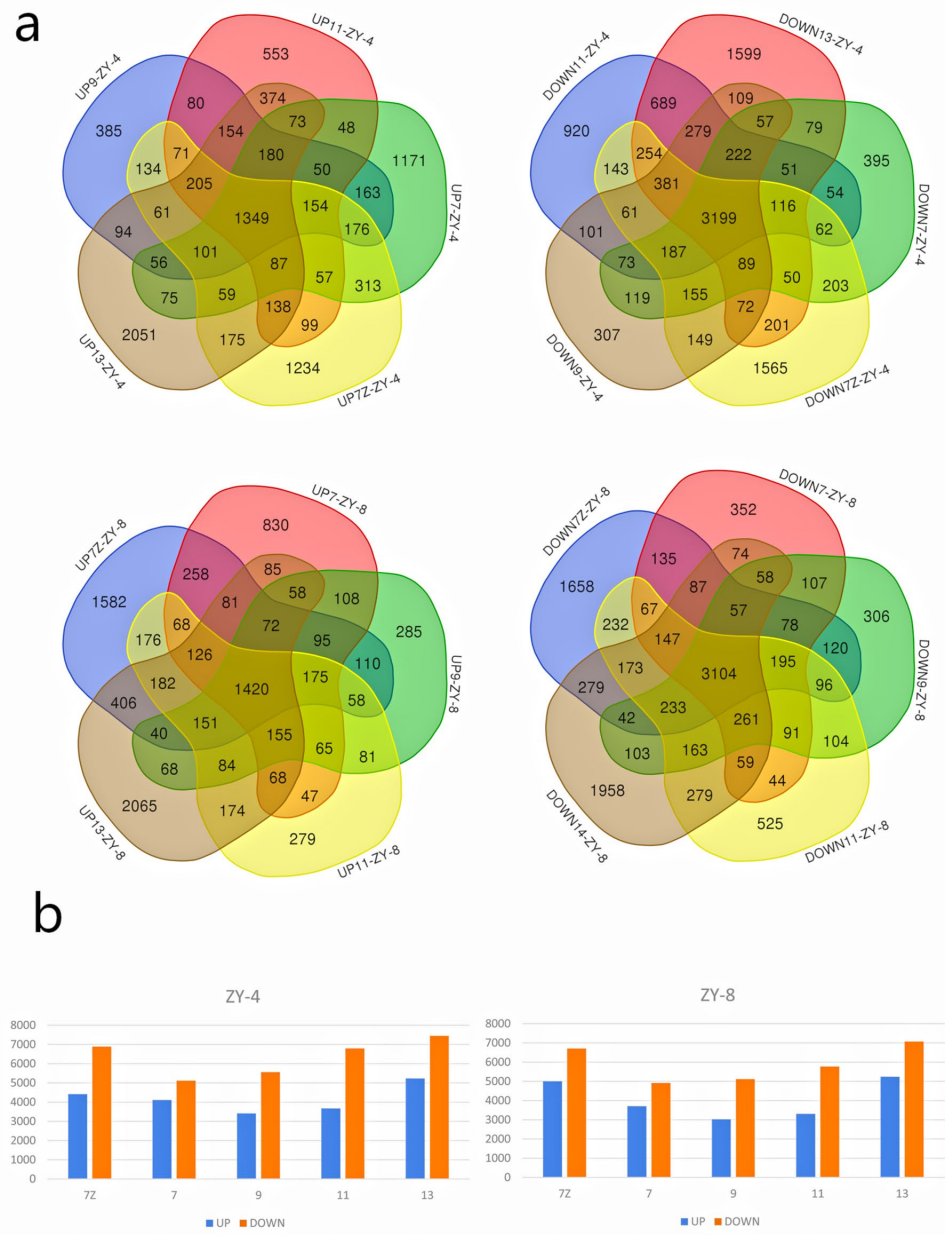
Differentially expressed genes (DEGs) in ZY-4 and ZY-8 at five seedling development stages. **a** Venn diagrams for the number of up- or down- DEGs at five seedling development stages. **b** The number of DEGs up- or down-regulated at five seedling development stages

A total of 21,379 and 20,322 DEGs were identified in ZY-4 and ZY-8 during all stages (Table S2, *P* value ≤ 0.01, *q* ≤ 0.01), respectively. Within the DEGs, there were 1349 same up-regulated genes and 3199 same down-regulated genes in ZY-4 at every stage. In ZY-8, there were 1420 same up-regulated genes and 3104 same down-regulated genes at every stage (Fig. 4a). In both mutants, downregulated genes were more than upregulated genes at every stage (Fig. 4b). The downregulated genes during all five stages were also more than the upregulated genes. We also compared up- or down-DEGs in ZY-4 and ZY-8 at every seedling stage and found many distinct DEGs between ZY-4 and ZY-8.

### GO and KEGG enrichment analysis of DEGs

**Fig. 5:**
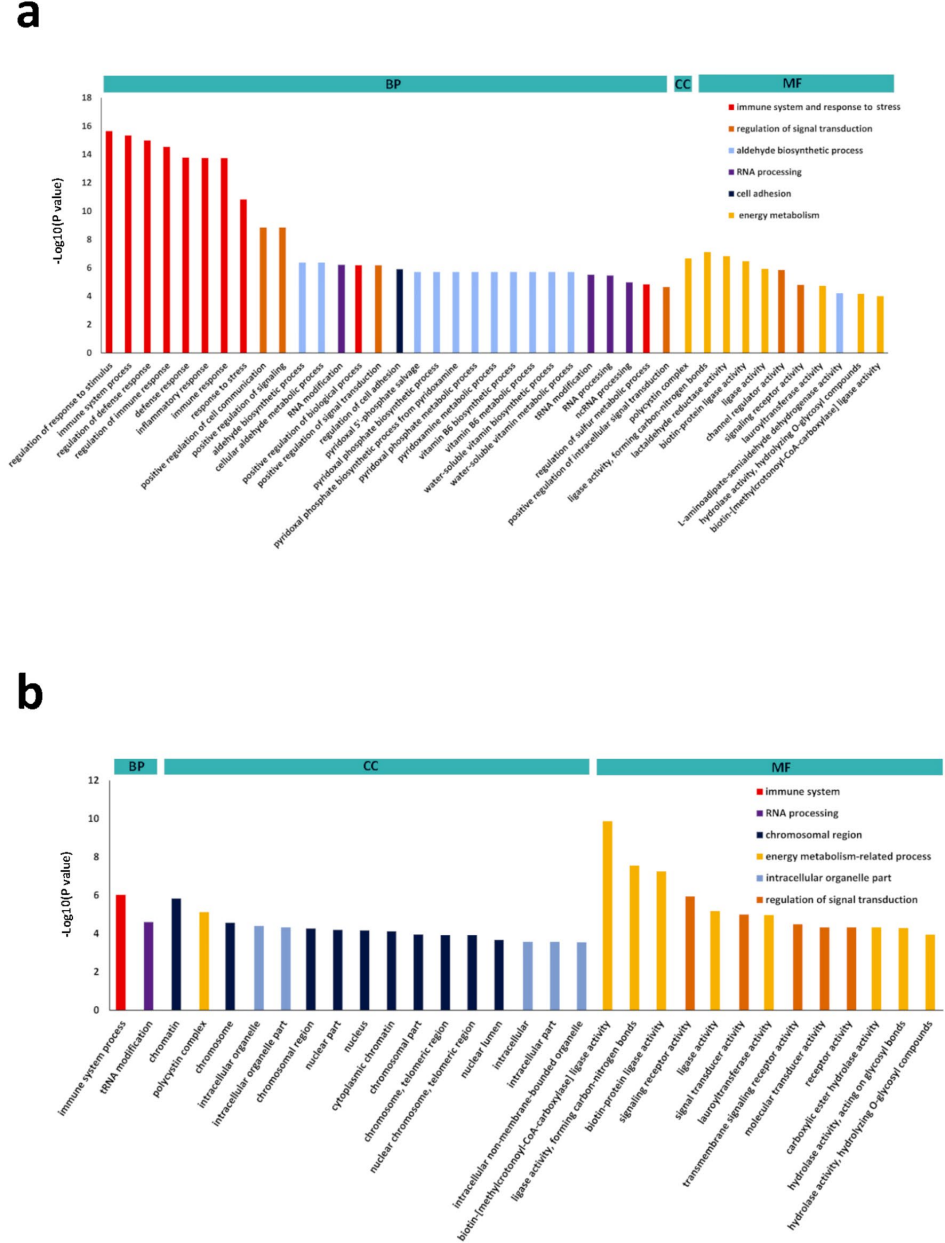
Analysis of GO enrichments for ZY-4 (**a**) and ZY-8 (**b**) showed that immune system process was the primary difference between mutants and ZS11. *P* value ≤ 0.01, q ≤ 0.01. *BP* biological process; MF, molecular function; CC, cellular component

Within the ZY-4 DEGs, 19,169 genes were annotated from at least one GO term, and assigned to 72 main GO terms (*P* value ≤ 0.01, *q* ≤ 0.01). In ZY-8 there were 18,162 DEGs annotated from at least one GO term and assigned to 31 main GO terms (*P* value ≤ 0.01, *q* ≤ 0.01). DEGs in ZY-4 were enriched in more GO terms, indicating that the ZY-4 gene expression pattern had additional differences with ZS11. We classified the GO terms into three different groups: biological process (BP), molecular function (MF) and cellular component (CC), as shown in the Fig. 5; details are listed in Table S3.

In ZY-4, the BP part contained the most GO terms (60). Only the top 30 BP terms were shown in Fig. 5a, all GO terms are listed in Table S3-1. ZY-8 had the smallest number of BP terms. BP terms in ZY-4 were put into these categorizes: immune system and response to stress (19), RNA processing (14), aldehyde biosynthetic process (12), regulation of signal transduction (8), and cell adhesion (8). There were eleven terms of the MF part in ZY-4, of which three terms were related to regulation of signal transduction, and one term was related to aldehyde biosynthetic processing. Seven other MF terms and one CC term were related to energy metabolism.

In ZY-8, there were sixteen GO terms related to energy metabolism (9), regulation of signal transduction (5), immune system (1), and RNA processing (1). Of these sixteen GO terms, 10 were also enriched in ZY-4, and the hits genes of each term in ZY-4 were same or more than which in ZY-8. Fifteen terms of the CC part in ZY-8 were related to chromosomal region (10) and intracellular organelle part (5).

In summary, DEGs in ZY-4 and ZY-8 were significantly enriched in the two biological processes: energy metabolism-related process, regulation of signal transduction. The “energy metabolism-related process” contained most of the enriched terms. ZY-8 was more similar to ZS11 than ZY-4 was to ZS11, with ZY-4 having more hits genes in the same GO terms, plus more enriched GO terms about same biological processes, especially “immune system and response to stress”. Further, DEGs in ZY-4 and ZY-8 were also enriched in different GO terms for different biological processes; the “aldehyde biosynthetic process” and “cell adhesion” in ZY-4, and the “chromosomal region” and “intracellular organelle part” in ZY-8.

**Fig. 6:**
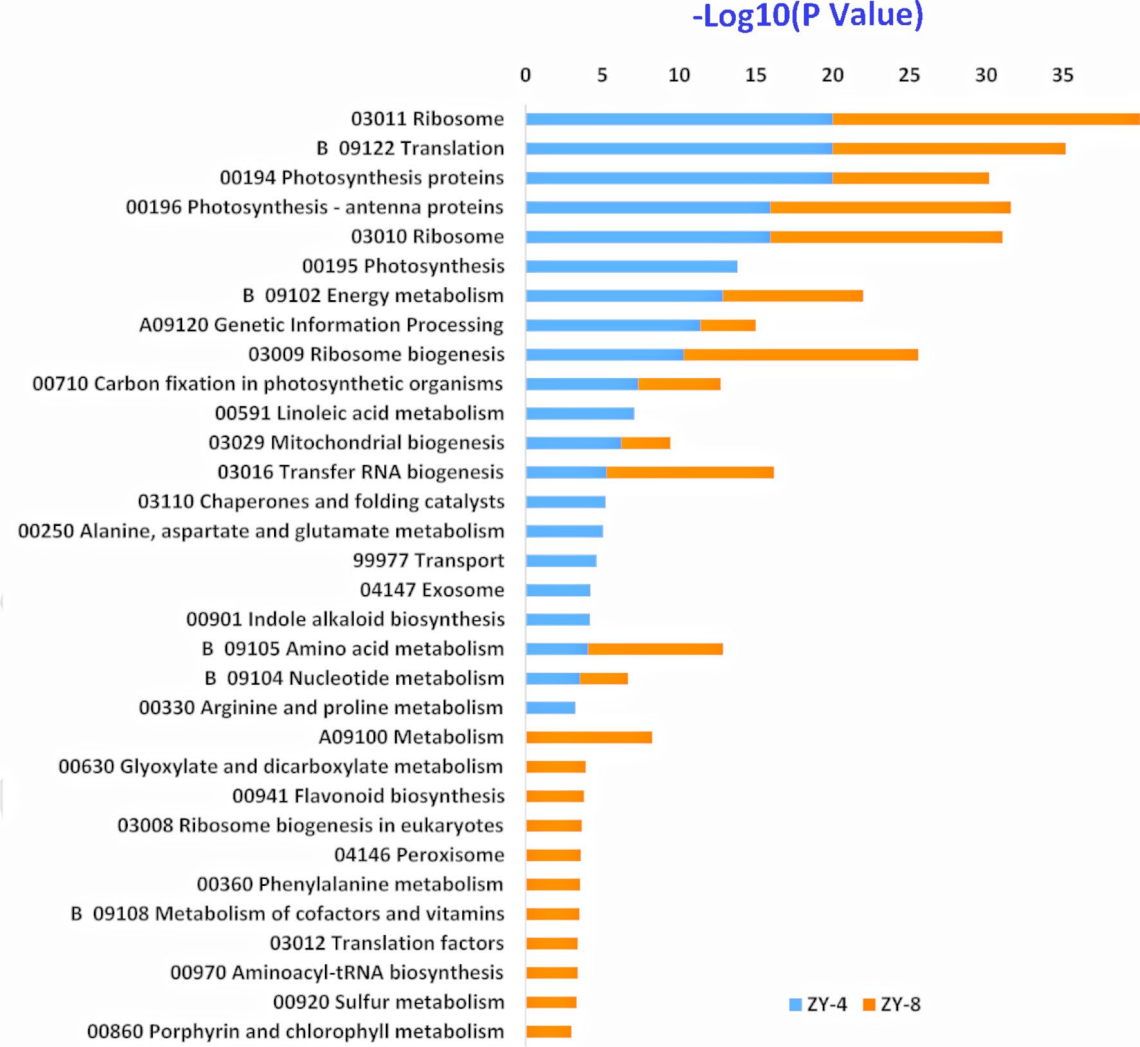
KEGG pathways for ZY-4 and ZY-8 showed that photosynthesis and energy metabolism-related pathways, plus translation related pathways, are important for the leaf variegation in the two mutants. *P* value ≤ 0.01, *q* ≤  0.01

To uncover the special biological pathways that were disturbed in ZY-4 and ZY-8, we mapped DEGs to the reference canonical pathways in the KEGG database. The DEGs of ZY-4 and ZY-8 enriched significantly in twenty one and twenty four pathways, respectively (Tables S3-3, S3-4, Fig. 6, *P* value ≤ 0.01, *q* ≤ 0.01). We found overlap between the enriched pathways in ZY-4 and ZY-8. Theses pathways were mainly about photosynthesis, energy metabolism, and translation. Two functional hierarchies about genetic information processing, “nucleotide metabolism” and “transfer RNA biogenesis” were also enriched in both mutants. While the biological objects within this category, “chaperones and folding catalysts” and “translation factors” were only enriched in ZY-4 and ZY-8, respectively. Moreover, “amino acid metabolism” was enriched in ZY-4 and ZY-8, “alanine, aspartate and glutamate metabolism”, “arginine and proline metabolism” were enriched in ZY-4, “phenylalanine metabolism” was enriched in ZY-8. Three other pathways significantly enriched in ZY-4 were “transport”, “exosome”, and “indole alkaloid biosynthesis”. Five other pathways enriched in ZY-8 were “metabolism”, “flavonoid biosynthesis”, “peroxisome”, “metabolism of cofactors and vitamins”, and “porphyrin and chlorophyll metabolism”.

Based on the GO and KEGG analysis, we concluded that photosynthesis and energy metabolism-related pathways, plus translation related pathways, were important for the leaf variegation in both mutants. Additionally, the different degrees of variegation between ZY-4 and ZY-8 might be related to the DEGs enriched in the immune system process.

### Comprehensive analysis of the enriched genes involved with photosynthesis, translation, and immune system process

**Fig. 7:**
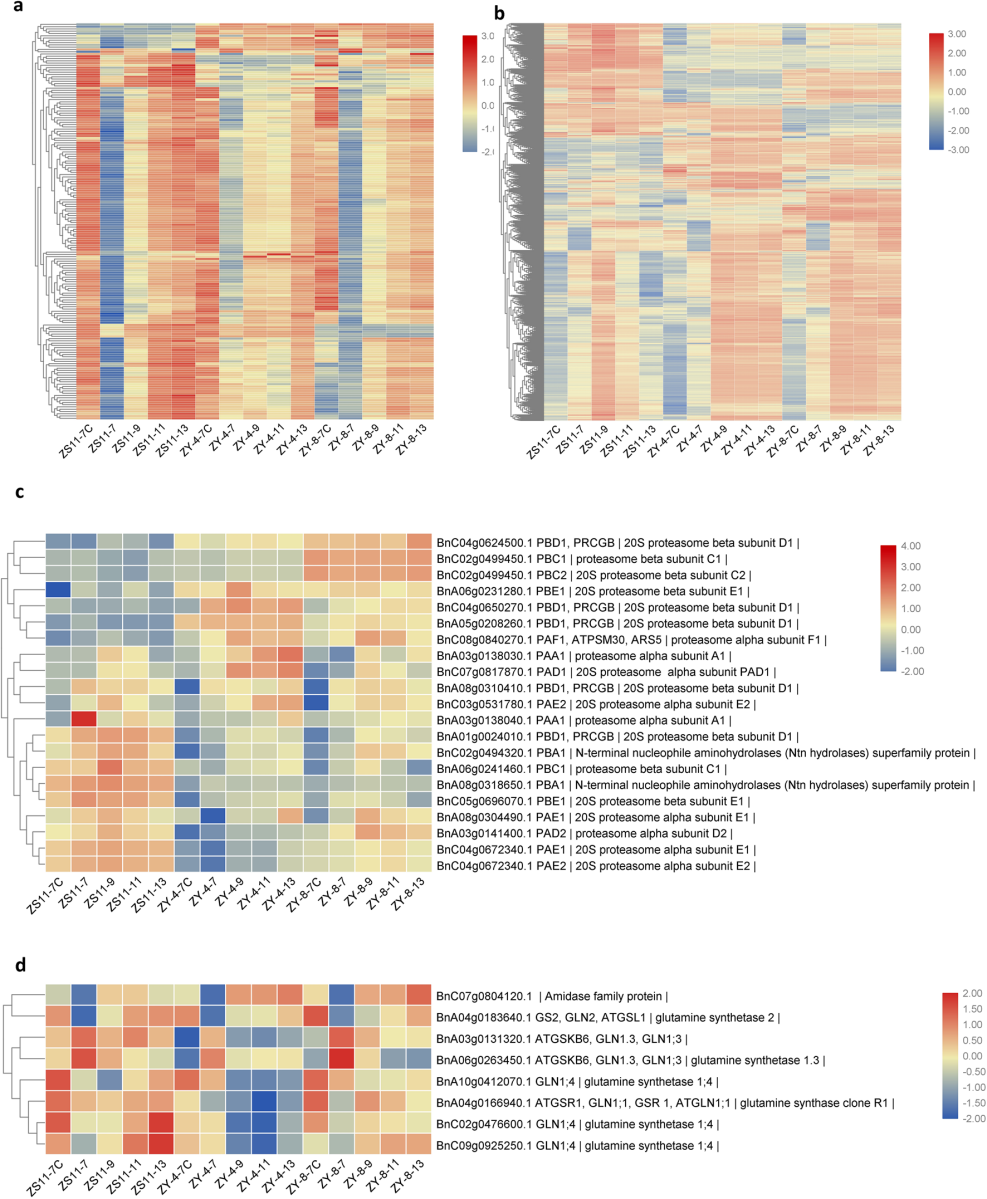
Heatmap showing the expression profiles of DEGs of photosynthesis genes (**a**), translation-related genes (**b**), 20S proteasome subunits genes (**c**), glutamine synthetase genes (**d**). **a** After 9DAS, the expression levels in two mutants were always downregulated compared with ZS11. **b** The expression levels of a significant number of translation-related genes did not decrease correspondingly after as ZS11 but maintained high expression levels after 9DAS. **c** The expression level of 20S proteasome subunits genes showed an opposite pattern between ZS11 and mutants. **d** Eight copies of glutamine synthetase genes were down-regulated to some extent in the two mutants; whereas in ZY-4, they decreased more than in ZY-8. *DAS* days after seeded

As a result of KEGG, “photosynthesis proteins” were enriched in both mutants with highly significant P values. This pathway is obviously important for chloroplast structure and function. We analyzed 174 genes enriched in this pathway to explore the differences between the mutants and ZS11 (Table S4-1, Fig. 7a) and found that most enriched genes were upregulated at 7 DAS in top meristems, with the highest expression levels in ZY-4. At 9 DAS seedlings, these photosynthesis protein genes were upregulated compared with 7 DAS, but the transcriptome pattern changed and many genes displayed different expression trends. After 9 DAS, the expression levels in two mutants were always downregulated compared with ZS11.

Genes related to translation process are mainly ribosomal subunit genes, translation initiation factor genes, translation elongation factor genes, ribosome recycling factor genes, rRNA and aminoacyl-tRNA synthesis, and modification genes, involved all translation processes. In analysis of gene expression related to translation processes, we found a significant number of translation-related genes with peak transcript abundances at 9 DAS (Table S4-2, Fig. 7b). The expression levels in cotyledons at 7 DAS and leaves at 13 DAS were relatively low. Therefore, we believed that these genes were early chloroplast development genes, with expression level associated with chloroplast development. In both mutants, the expression levels of these genes did not decrease correspondingly but maintained high expression levels after 9 DAS. In both mutants, early chloroplast development genes continued to be expressed.

Concerning the immune system and response to stress, GO terms enriched in ZY-4 were from two to six GO levels, however, ZY-8 was only enriched in the level two GO term “immune system process”. Under this term, there were 562 and 466 genes enriched in ZY-4 and ZY-8, respectively. We analyzed the 318 overlap genes between ZY-4 and ZY-8. These genes primarily include ribosome and translation related genes, tRNA lineage and modification genes, and photosynthesis genes. The translation related genes and photosynthesis genes were previously analyzed in this study. Therefore, we were interest in the genes enriched in the immune process. The expression level of seven 20S proteasome subunits genes showed an opposite pattern between ZS11 and mutants. The expression level of BnC06g0770560.1 in ZY-8 was between ZS11 and ZY-4. Interestingly, six enriched genes reached the highest levels at 9 DAS, with the expression levels decreasing in ZS11 but not in mutants (Table S4-3, Fig. 7c). In the enriched genes of the immune system, there were eight copies of glutamine synthetase genes. They were down-regulated to some extent in mutants; whereas, they decreased in ZY-4 more than in ZY-8 (Table S4-4, Fig. 7d).

### Differences in the expression of key genes expression related to chloroplast-to-nucleus retrograde signaling pathways and plastoglobule-specific proteins

Chloroplast-to-nucleus retrograde signaling pathways are extremely important for seedlings. The development of functional chloroplast relies on the coordination of expressions of both nuclear and chloroplast genomes. The mis-regulation of photosynthetic development can lead to severe photooxidative damage and seedling mortality (Chan et al. 2016). The structures of chloroplasts in ZY-4 and ZY-8 were damaged, and the GO and KEGG pathway analyses suggested that many functions of chloroplasts were disrupted. We analyzed the expression of the genes in the retrograde pathway to explore the differences between the mutants and ZS11.

**Fig. 8:**
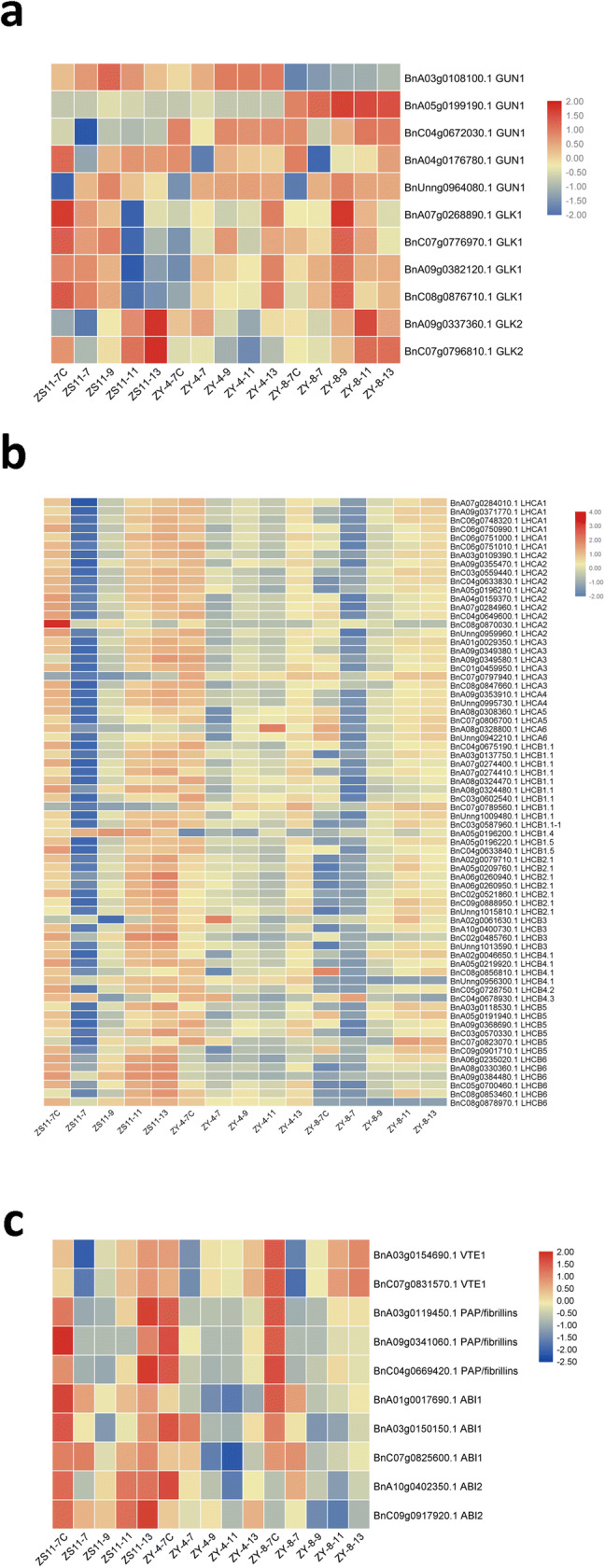
Heatmap showing the expression profiles of chloroplast-to-nucleus retrograde signaling pathway genes (**a**), antenna protein genes (**b**), plastoglobule-specific proteins (**c**). **a** The expressing pattern of GUN1 in ZY-8 was more different to ZS11. The expression levels of the four of GLK1 decreased significantly within 11 days, while the GLK2 levels gradually increased with the growth of the plant. **b** In ZY-4, the expression levels of antenna protein genes were not increasing with the growth, and the expression levels were higher than ZS11 only for 7 DAS true leaf. The expression of LHC subunits in ZY-8 were lower than in ZS11 during the five development stages, but the expression patterns were similar to ZS11. **c** The plastoglobules observed in the ZY-4 and ZY-8 were not induced by the expression of the plastoglobule-specific protein genes

GUN1 is a central integrator of chloroplast retrograde signals that relates to the expression of the nuclear genes (Brunkard and Burch-Smith 2018). We found six copies of GUN1 in ZS11, five of which were expressed in ZY-4, ZY-8 and ZS11. Interestingly, compared to ZY-4, the expressing pattern of GUN1 in ZY-8 was more different to ZS11 (Table S4-1, Fig. 8a), especially the expression of BnA03g0108100.1 and BnA05g0199190.1 was down-regulated and up-regulated, respectively. In ZY-4, the expressing pattern of BnA05g0199190.1 did not have any obvious differences with ZS11, which was almost not expressed. Another copy of GUN1, BnC04g0672030.1, was both up-regulated in ZY-4 and ZY-8. BnA04G0176780.1 had similar expression levels in mutants and ZS11, with lowest expression level in top meristem at 7 DAS. Interestingly, BnA03g0108100.1 and BnUnng0964080.1 had similar expression patterns with peak transcript abundance at 9 DAS in ZS11. In ZY-8, the expression level of BnA03g0108100.1 was very low, while the expression pattern of BnUnng0964080.1 was similar to ZS11. In ZY-4, the expression levels of these two genes maintained relatively high levels at 9 DAS, 11DAS and 13 DAS.

Further, we wanted to check the expression of the genes at the downstream of GUN1. GOLDEN 2-LIKE1 (GLK1) and GLK2 are two MYB transcription factors at downstream of GUN1 that promote Photosynthetic Associated Nuclear Genes (PhANG) expression (Brunkard and Burch-Smith 2018). There are four copies of GLK1 and two copies of GLK2 in ZS11. The expression levels of the four copies of GLK1 decreased significantly within 11 days; while, the GLK2 levels gradually increased with the growth of the plant (Table S5-1, Fig. 8a). In ZY-4, the expression level of GLK1 peaked at 13 DAS; while in ZY-8, the peak level appeared at 9DAS. The expression of GLK2 was downregulated in ZY-4 and ZY-8 during the early stages of the seedling development, but in ZY-8, the expression level of GLK2 began to rise at 11 DAS. Constitutive GLK gene expression leads to increased accumulation of transcripts for light-harvesting antenna proteins and chlorophyll biosynthetic enzymes (Waters et al. 2009). We checked the expression levels of all antenna proteins genes in ZS11, ZY-4 and ZY-8. There are 72 genes in the ZS11 homologous with antenna genes, 70 of which expressed in ZS11 seedlings (details listed in the Table S5-2 and Fig. 8b). The antenna proteins of light-harvesting complex I (LHC I) and LHC II had similar expression patterns. In the 70 homologous genes of the LHC I and LHC II subunits, we found that transcription levels of the ZS11 LHC subunits were very low in top shoot at 7DAS, increasing with the seedling growth from 7 to 13 DAS. The expression level of 7 DAS cotyledon was very high, even higher than that of 13 DAS true leaf. Interestingly, in ZY-4, the expression levels of antenna protein genes were not increasing with the growth, and the expression levels were higher than ZS11 only for 7 DAS true leaf. 7 DAS cotyledon had the highest expression levels of LHC antenna genes. The expression of LHC subunits in ZY-8 was lower than in ZS11 during the five development stages, but the expression patterns were similar to ZS11, which increased with growth.

Oxidative stress has been shown to increase plastoglobule numbers and produce plastoglobuli clusters in chloroplasts. Oxidative stress can also lead to increases in plastoglobule-specific proteins, including VTE1 and PAP/fibrillins. It has been suggested that plastoglobuli formation is dependent on the synthesis of such proteins (Austin et al. 2006). In ZY-4 and ZY-8, the expression of these two genes did not noticeably increase compared with ZS11 (Table S5-3, Fig. 8c), indicating that the plastoglobules in the chloroplasts of mutants did not depend on the expression of VTE1 and PAP/fibrillins. VTE1 is a major limiting factor of tocopherol synthesis in leaves, which has been related to moderating oxidative stress (Kanwischer et al. 2005). In our study, the expression levels of VTE1 from high to low were ZY-8, ZS11, ZY-4. The expression of PAP/fibrillins in ZS11 increased at 13 DAS, but did not obviously increase in ZY-4 and ZY-8. We checked the expression of ABI1 and ABI2, which regulate PAP/fibrillin expression in Arabidopsis (Yang et al. 2006), and found three copies of ABI1 and two copies of ABI2 that did not have the same expression patterns with PAP/fibrillin genes. In the two variegation mutants, ABA response regulators did not activate the expression of PAP/fibrillin. Our results demonstrated that the plastoglobules observed in the ZY-4 and ZY-8 were not induced by the expression of the plastoglobule-specific protein genes.

### Identification of candidate genes

As a result of the SNP analyses of the BC_2_F_1_ lines from ZY-4 and ZS11, plus ZY-8 and ZS11, there were two loci in ZY-4 at A08 and C04, one locus in ZY-8 at A08, related to the variegation phenotype. The candidate regions at A08 overlapped in ZY-4 and ZY-8. We analyzed the expression of genes in the two candidate regions. The expression level of these genes is listed in the Table S5.

There were 902 genes in the 10–15-Mb region of A08 (Table S6-1), of which 662 were expressed during the five seedling development stages and 125 expressed genes proteins were chloroplast protein. Among the 125 genes, 19 were upregulated in ZY-4 and ZY-8, and nine were downregulated in ZY-4 and ZY-8 (Table 1, Table S6-2). The putative functions of these genes were adopted from their orthologs in *Arabidopsis thaliana*.

**Table 1:** Candidate genes for variegation of ZY-4 and ZY-8

Gene ID	Up or down regulated in ZY-4	Up or down regulated in ZY-8	Ortholog in *Arabidopsis. thaliana*	Subcellular localization	Description
BnaA08g17820D	Down	Down	AT1G29670.1	Chloroplast thylakoid	GDSL1:GDSL-motif esterase/acyltransferase/lipase
BnaA08g11590D	Down	Down	AT4G34120.1	Chloroplast stroma	CDCP1: Cystathionine beta-synthase (CBS) family protein
BnaA08g14980D	Down	Down	AT4G35460.1	Chloroplast envelope	NADPH-dependent thioredoxin reductase 1
BnaA08g11840D	Down	Down	AT4G33300.2	Chloroplast	ADR1-L1: a member of the ADR1 family immune receptors
BnaA08g14220D	Down	Down	AT4G26970.1	Chloroplast	ACO2 aconitase 2; 4 iron, 4 sulfur cluster binding
BnaA08g16720D	Down	Down	AT4G39110.1	Chloroplast	BUPS1: regulation of pollen tube growth
BnaA08g17160D	Down	Down	AT4G14210.2	Chloroplast	PDS3: Encodes phytoene desaturase (phytoene dehydrogenase) biosynthesis
BnaA08g17170D	Down	Down	AT4G14210.1	Chloroplast	PSD3: Encodes phytoene desaturase (phytoene dehydrogenase) biosynthesis
BnaA08g18100D	Down	Down	AT1G29195.1	Chloroplast	Phosphatidylinositol 4-phosphate 5-kinase MSS4-like protein
BnaA08g13300D	Up	Up	AT4G29840.1	Chloroplast stroma	MTO2: threonine synthase
BnaA08g11450D	Up	Up	AT4G33760.1	Chloroplast	OKI1: Aminoacyl tRNA Synthetase functions in SAM maintenance
BnaA08g11470D	Up	Up	AT4G33680.1	Chloroplast	AGD2: systemic acquired resistance, salicylic acid mediated signaling pathway
BnaA08g11490D	Up	Up	AT4G33650.1	Chloroplast	DRP3A: Involved in peroxisome and mitochondria fission in combination with DRP3B
BnaA08g12180D	Up	Up	AT4G32520.1	Chloroplast	SHM3: tetrahydrofolate metabolic process
BnaA08g12530D	Up	Up	AT4G31370.1	Chloroplast	FLA5: fasciclin-like arabinogalactan-protein, putative (FLA5)
BnaA08g13690D	Up	Up	AT4G28510.1	Chloroplast	PHB1: mitochondrial respiratory chain complex I
BnaA08g14500D	Up	Up	AT4G25300.1	Chloroplast	Oxidation–reduction process 2-oxoglutarate (2OG) and Fe(II)-dependent oxygenase superfamily protein
BnaA08g14510D	Up	Up	AT4G25300.2	Chloroplast	Oxidation–reduction process 2-oxoglutarate (2OG) and Fe(II)-dependent oxygenase superfamily protein
BnaA08g14850D	Up	Up	AT4G35890.1	Chloroplast	LARP1C: a cytoplasmic LAM domain containing protein that is involved in leaf senescence
BnaA08g16710D	Up	Up	AT4G39120.1	Chloroplast	IMPL2: inositol monophosphate 4-phosphatase activity
BnaA08g16930D	Up	Up	AT4G38730.1	Chloroplast	AVI2H: integral component of membrane magnesium transporter
BnaA08g17110D	Up	Up	AT4G38380.1	Chloroplast	MATE efflux family protein
BnaA08g18260D	Up	Up	AT1G28530.2	Chloroplast	ANU10: a protein is required for both chloroplast and mesophyll development
BnaA08g18660D	Up	Up	AT1G27980.1	Chloroplast	DPL1: dihydrosphingosine phosphate lyase
BnaA08g18950D	Up	Up	AT1G27460.1	Chloroplast	NPGR1: a calmodulin-binding protein that is expressed in pollen
BnaA08g18960D	Up	Up	AT1G27450.1	Chloroplast	APT1: circadian rhythm Adenosine phosphoribosyl transferase
BnaA08g19610D	Up	Up	AT1G26180.1	Chloroplast	Chloroplast membrane protein
BnaA08g20080D	Up	Up	AT1G27090.1	Chloroplast	mRNA binding glycine-rich protein
BnaC04g34600D	Down	–	AT5G42070.1	Chloroplast	–

In the genes both downregulated in ZY-4 and ZY-8, BnaA08g17160 and BnaA08g17170D were highly orthologous to *AtPDS3* (AT4G14210). AT4G14210 encodes phytoene desaturase, an enzyme that catalyzes the desaturation of phytoene to zeta-carotene during carotenoid biosynthesis (Pecker et al. 1992; Scolnik and Bartley 1993). Mis-splicing of the PDS3 RNA transcript obtained albino phenotypes (Kang et al. 2018). PDS3 silencing also can produce a mottled photobleaching phenotype in Arabidopsis, tobacco, wheat, and barley (Wang et al. 2005; Cai et al. 2006; Montgomery et al. 2008; Yuan et al. 2011; Jiang et al. 2013; Tian et al. 2014; Ma et al. 2015a). The white sectors of the Arabidopsis variegated mutant *immutans* contain abnormal chloroplasts that lack colored carotenoids due to a defect in phytoene desaturase activity (Carol et al. 1999; Wu et al. 1999; Aluru et al. 2009). As the phenotype of ZY-4 and ZY-8 was similar to *immutans*, we speculated that the carotenoid biosynthesis pathways were disrupted in ZY-4 and ZY-8, and checked the expression of genes in carotenoid biosynthesis pathway. The carotenoid biosynthesis pathway is well established, beginning with the formation of C5 IPP and DMAPP via MEP pathway. PSY is the “bottleneck” enzyme that affects carotenoid pool size, and there are six copies of PSY in *B. napus*. Compared to a true leaf, the expression levels of PSY in cotyledons of ZS11, ZY-4 and ZY-8 were higher at 7 DAS, and ZY-8 was higher than ZS11 and ZY-4. In ZS11, the level of PSY transcription increased with leaf development, but in ZY-4 and ZY-8, the expression patterns of PSY were not properly regulated, resulting in PSY being completely downregulated. Following a series of desaturation and isomerization reactions catalyzed by phytoene desaturase (PDS), ζ-carotene desaturase (ZDS), ζ-carotene isomerase (Z-ISO), and carotenoid isomerase (CRTISO), a carotenoid lycopene is produced (Nisar et al. 2015; Sun et al. 2018). There are five copies of PDS in ZS11, and each were expressed in all five stages, with three copies (BnaC03g76050D, BnaC04g28970D, and BnaA04g06150D) having high expression levels. In ZY-4 and ZY-8, three copies (BnaC04g28970D, BnaA08g17160D, and BnaA08g17170D) were downregulated. BnaA04g06150D was downregulated in ZY-4 and ZY-8, but the expression level in ZY-4 was higher than in ZY-8. In general, PSY and PSD were downregulated in ZY-4 and ZY-8. These two genes are very important for carotenoid metabolism in plants, an indication that the carotenoid biosynthesis pathway was disrupted in ZY-4 and ZY-8.

There were 80 genes in 36–37-Mb interval of C04 (Table S6-3), of which 41 were expressed during the seedling development stages (Table S6-4). The subcellular locations of nine expressed genes were chloroplasts. Of these nine genes, one gene (BnaC04g34600) was downregulated only in ZY-4 (Table 1); no gene was upregulated only in ZY-4. BnaC04g34600 is orthologous to AT5G42070 in Arabidopsis, and Bra028524 in *Brassica rapa.* Bra028524 was upregulated in the *drm* mutant (a slow-growing reduced-thylakoid mutant of Chinese Cabbage) at three leaves visible stage (Huang et al. 2016). BnaC04g34600 may be related to the thylakoid generation and may influence ZY-4 phenotype.

### Superoxide anion accumulation in the ZS11, ZY-4 and ZY-8

**Fig. 9:**
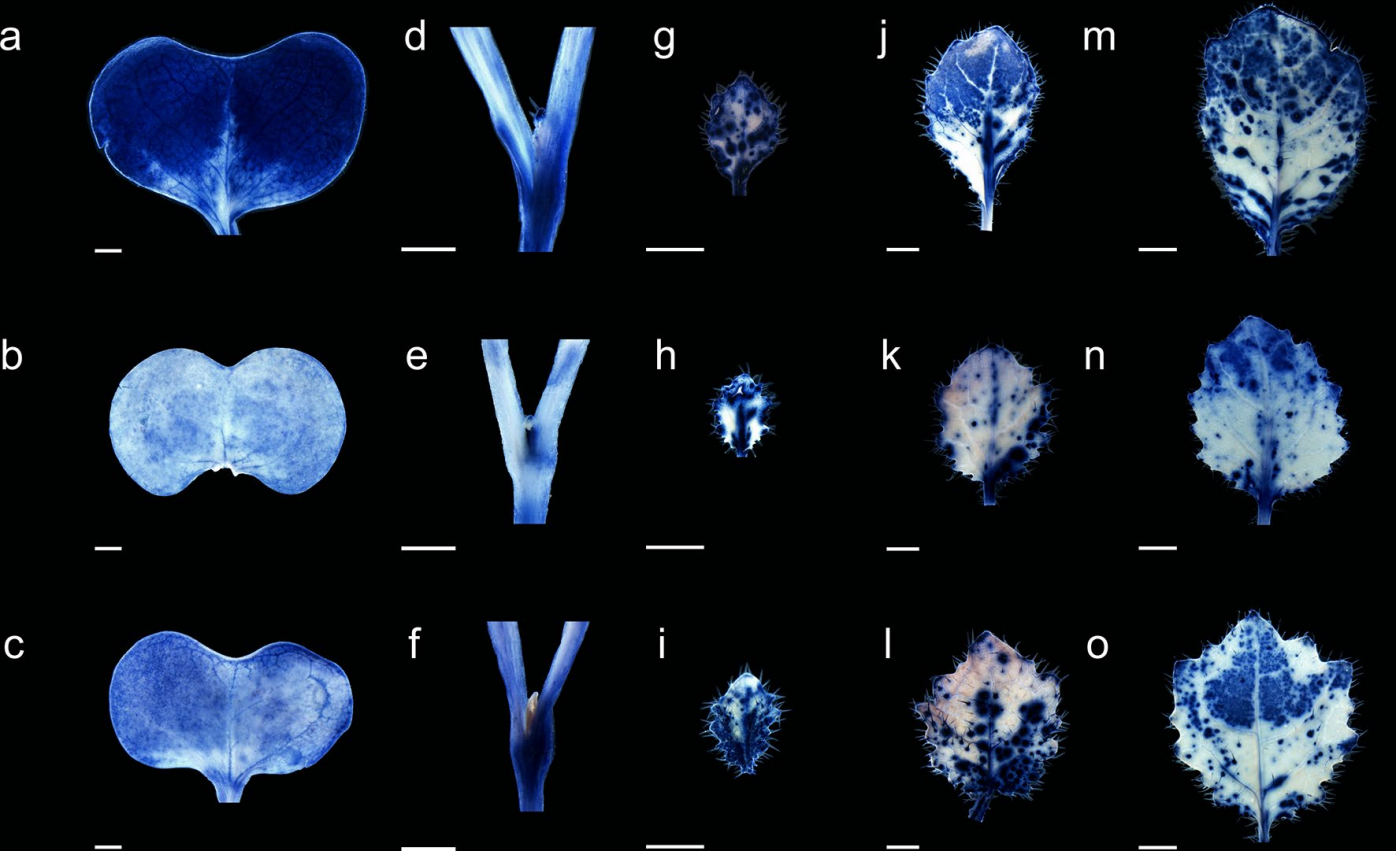
Superoxide anion accumulation in the ZS11 (**a**, **d**, **g**, **j**, **m**), ZY-4 (**b**, **e**, **h**, **k**, **n**) and ZY-8 (**c**, **f**, **i**, **l**, **o**). Cotyledon of 7 DAS (**a**–**c**), top shoot of 7 DAS (**d**–**f**), first true leaf of 9 DAS (**g**–**i**), first true leaf of 11 DAS (**j**–**l)**, first true leaf of 13 DAS (**m**–**o**). Compared with ZS11, there was no excessive accumulation of ROS in the true leaves of the two mutants. *DAS* days after seeded. Scale bar 2 mm

In the chloroplasts of ZY-4 and ZY-8, we observed plastoglobuli. The thylakoid membranes were first generated, then degraded or destroyed in ZY-4 and ZY-8. According to the result of the candidate genes analyses, carotene biosynthetic pathways were disrupted in ZY-4 and ZY-8. Colored carotenoids protect chloroplasts against ROS-induced photooxidation by quenching triplet chlorophyll and singlet oxygen (DemmigAdams and Adams 1996; Young and Frank 1996; Ruban et al. 2007), suggesting that the plastids in ZY-4 and ZY-8 white sectors are photooxidized. As a result of unbalanced metabolic reactions, ROS were rapidly formed, and many found to be produced inside chloroplasts, yielding lipid peroxides and H_2_O_2_ that could damage thylakoid proteins (Awad et al. 2015). We believe the abnormal biogenesis of chloroplasts in ZY-4 and ZY-8 seedling cause the generation of excessive ROS. As a result of ROS damaging thylakoid structures, ZY-4 and ZY-8 develop variegation phenotypes. To verify this hypothesis, we analyzed the superoxide anion accumulation in ZY-4 and ZY-8. Using nitroblue tetrazolium (NBT) staining, which indicated of ^1^O_2_ production, in ZY-4 cotyledons, the ROS accumulation was the lowest, and in ZS11 cotyledons, it was the highest. Compared with ZS11, there was no excessive accumulation of ROS in the true leaves of the two mutants (Fig. 9).

## Discussion

Photosynthesis genes are regulated by the developmental status of a chloroplast (McCormac and Terry 2004; Koussevitzky et al. 2007; Woodson et al. 2011; Chan et al. 2016; Martin et al. 2016; Page et al. 2017). At 7 DAS, ZY-4 had high transcription levels of photosynthesis genes. The expression level of these genes was always downregulated at all stages in this study, even though ZY-4 had increasing expression levels. Thus, the expression levels of photosynthesis genes were upregulated in true leaves at 7 DAS, but as the leaves continued developing, the upregulated trend was not obvious, and the expression of photosynthesis genes was suppressed to some extent. In ZY-8, photosynthesis genes were suppressed to a lesser degree. The conversion of proplastids into chloroplasts is accompanied by high transcription levels of plastid- and nuclear-encoded genes involved in the transcription/translation apparatus (Baumgartner et al. 1989). The expression of these genes decreases once the mature chloroplast is established (Mache et al. 1997; Gutierrez-Nava Mde et al. 2004). But in ZY-4 and ZY-8, the expression of these genes was not decreased when the chloroplasts in ZS11 were mature. We believe that the development of chloroplast was inhibited in ZY-4 and ZY-8, which affected the transcription of photosynthesis genes. However, we cannot explain the upregulation of photosynthesis genes in true leaves of mutants at 7 DAS. Additionally, the expression of GUN1 and GLK1/2 cannot explain the expression pattern of these genes at 7 DAS. The retrograde signaling pathways also displayed a disturbed network. Although the expression levels of photosynthesis genes were delayed in both mutants, as the expression levels increased with the development of the seedlings, leaves turned green gradually.

In chloroplasts, photosystems I and II (PSI and PSII) are the major sites for production of ^1^O_2_ and O_2_^−^, which are the major ROS involved in photooxidative lipid oxidation and membrane damage involved in galactolipid fragmentation (Foyer et al. 1994; Foyer and Noctor 2000; Triantaphylides et al. 2008; Gill and Tuteja 2010). As the ultrastructure of chloroplasts in the two mutants were destroyed in different extent, we considered whether the extensive ROS generated by abnormal chloroplasts were responsible for the chloroplast structure damages of chloroplast. However, the histochemical analysis of ROS found that the superoxide anion accumulation in the two mutants was lower than ZS11. Thus, we cannot link variegation directly to ROS accumulation.

The expression of retrograde signal pathway genes plus the increase in plastoglobule numbers indicate oxidative stress. In the analysis of RNA expression profiles, we found evidence showing ROS were related with the morphology of the two variegation mutants. The GO and KEGG analyses of transcriptomes showed that the “immune system process” was enriched to different extent in both mutants. ROS, as an important signal molecules, can induce immune processes (Nomura et al. 2012; Trotta et al. 2014). ROS may induce abnormal immune system gene expression, implying that two mutants were under oxidative stress. In the enriched immune system genes, there were many 20S proteasome genes and glutamine synthetase genes. In in vitro studies, it was found that 20S proteasome complex actively recognizes and degrades oxidized proteins (Davies 2001). The glutamine synthetase (GS), a key enzyme in photorespiration, assimilates ammonia into glutamine, which translocates organic nitrogen from sources to sinks within plants. High quantities of GS improve the capacity of plants for photorespiration and increased tolerance of high-intensity light (Kozaki and Takeba 1996; Lam et al. 1996; Foyer and Noctor 2000).The GO analyses of ZY-8 found 10 GO terms about chromosomal region. There were 166 genes enriched in this term, 43 of which were glutathione s-transferase, an important enzyme in antioxidant defense systems (Gill and Tuteja 2010). The KEGG result of ZY-8 showed many genes enriched in “flavonoid biosynthesis” and “peroxisome”, which are related to oxidant stress. The ROS, or redox state, of chloroplasts were affected; however, this did not necessarily destroy the chloroplasts as the mutants may be able to regulate other pathways to modulate ROS levels and reduce damage. ZY-8 had more enriched genes related to ROS, which were not in immune system. The degrees of variegation in ZY-8 was lower, which further indicates that mutants variegation is related to ROS.

Comparing the RNA-seq and BSA CHIP results found that homologous genes of PDS3, BnaA0817160D and BnaA0817170D, were in the candidate region and were downregulated in both mutants. The expression patterns of other copies of PDS3 were different and the expression level of PDS3 generally declined. Overexpression of *AtPDS* in tomato demonstrates *PDS* as a target for manipulation elevated lycopene content in maturing tomato fruit. *PDS* also constrains the ripening-induced PSY expression and activity (McQuinn et al. 2018). In ZY-4 and ZY-8, the different copies of PSY gene were generally downregulated. PSY and PDS are two most important genes in carotenoid biosynthetic pathways and we believe these pathways were not fully functional in both mutants. Carotenoids are essential components of photosynthesis and protect against photooxidative damage, and stabilize membrane (Walter and Strack 2011; Nisar et al. 2015; Sun et al. 2018). We support the idea that the disruption of carotenoids biosynthetic pathways contributes to the variegation phenotype in ZY-4 and ZY-8. We also found that different copies of PSY and PDS had different expression patterns. In normal green leaves of ZS11, the expression levels of different copies of PSY and PDS were different. In the variegated leaves of the mutants, the expression of different copies of PSY and PDS was also regulated differently. *Brassica napus* is an allopolyploidy species that evolved from a whole-genome triplication that was accompanied by gene expression divergence (Chalhoub et al. 2014; Dun et al. 2014). The different expression and regulation of different copies of PSY and PDS are models for studying the evolution of multi-copy genes and the effects of polyploidy.

Based upon BSA CHIP result, there may be two loci in ZY-4 that influenced the variegation. The candidate gene in A08, *PDS3*, is an important gene that leads to the variegation phenotype when downregulated. However, in C04 candidate region, only one gene, BnaC04g34600, was found to be downregulated only in ZY-4. This gene encodes a protein that locates in chloroplast according to homologous gene in Arabidopsis. To better explain the higher degree of variegation in ZY-4, more research is needed on ROS and photoprotection, to find relationship between the genes in C04 candidate region and variegation.

In this study, we found important genes and molecular pathways for the variegation of ZY-4 and ZY-8. Further efforts should be focused on the map-based cloning of mutant genes to determine if *PDS3* is the primary gene contributing to the variegation of ZY-4 and ZY-8.

## Electronic supplementary material

Below is the link to the electronic supplementary material.

Supplementary file1 (TIF 1051 kb)

Supplementary file2 (TIF 184 kb)

Supplementary file3 (XLSX 36 kb)

Supplementary file4 (XLSX 35902 kb)

Supplementary file5 (XLSX 5607 kb)

Supplementary file6 (XLSX 369 kb)

Supplementary file7 (XLSX 32 kb)

Supplementary file8 (XLSX 187 kb)

## Data Availability

The raw sequence data were deposited in the NCBI Sequence Read Archive (Accession No. PRJNA559661). The SRA records will be accessible with the following link after 2020/09/08: https://www.ncbi.nlm.nih.gov/sra/PRJNA559661. Reviewer link: https://dataview.ncbi.nlm.nih.gov/object/PRJNA559661?reviewer=blpt4mt03e1a62060cd51tr4i1.
