# Supplementary figures and images for: Disruption of carotene biosynthesis leads to abnormal plastids and variegated leaves in Brassica napus
Source: Mol Genet Genomics. 2020 Apr 18;295(4):981–99. doi: 10.1007/s00438-020-01674-w (PMC7297816; doi:10.1007/s00438-020-01674-w)

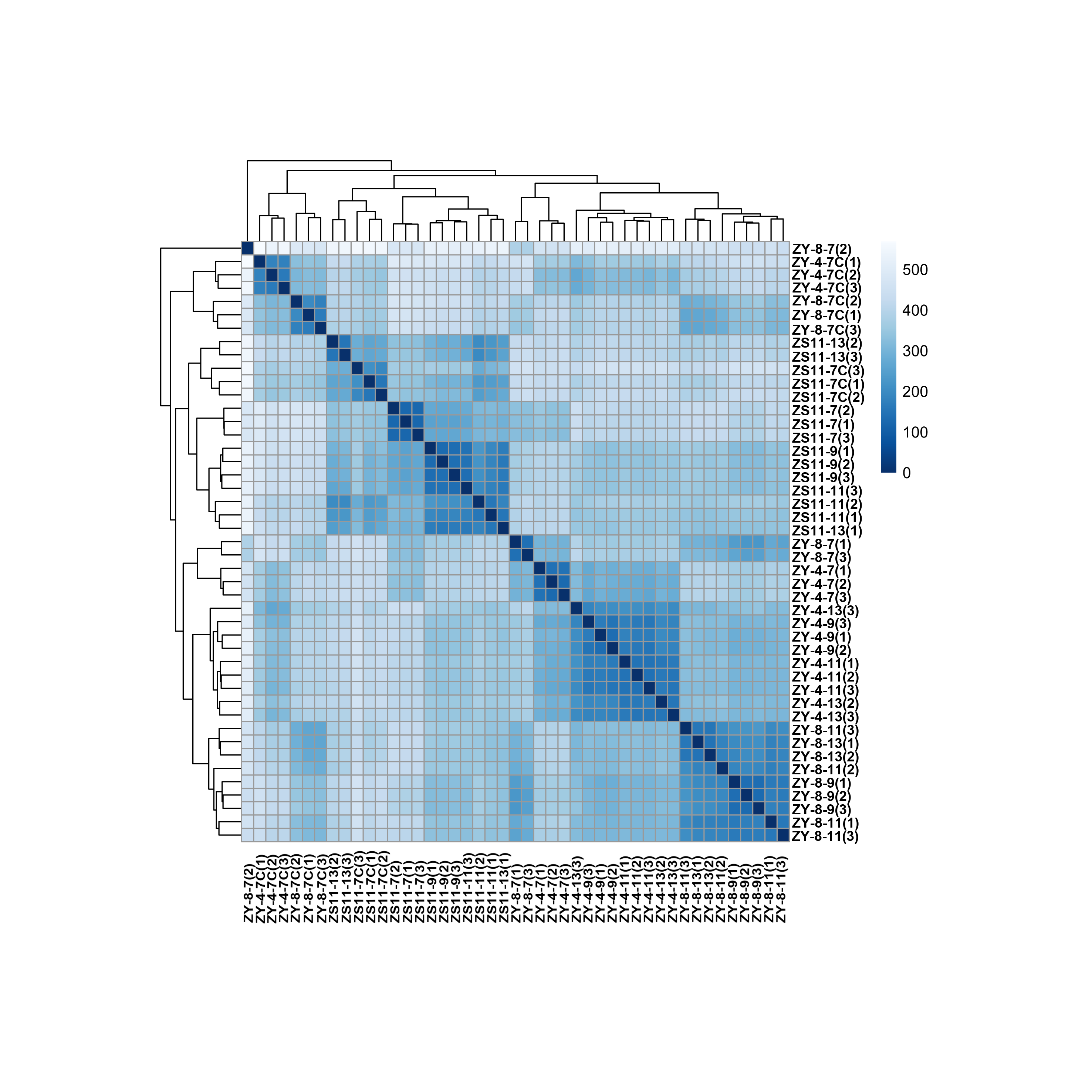

Supplement: Supplementary file 1 — Supplementary file1 (TIF 1051 kb) [file 438_2020_1674_MOESM1_ESM.tif]

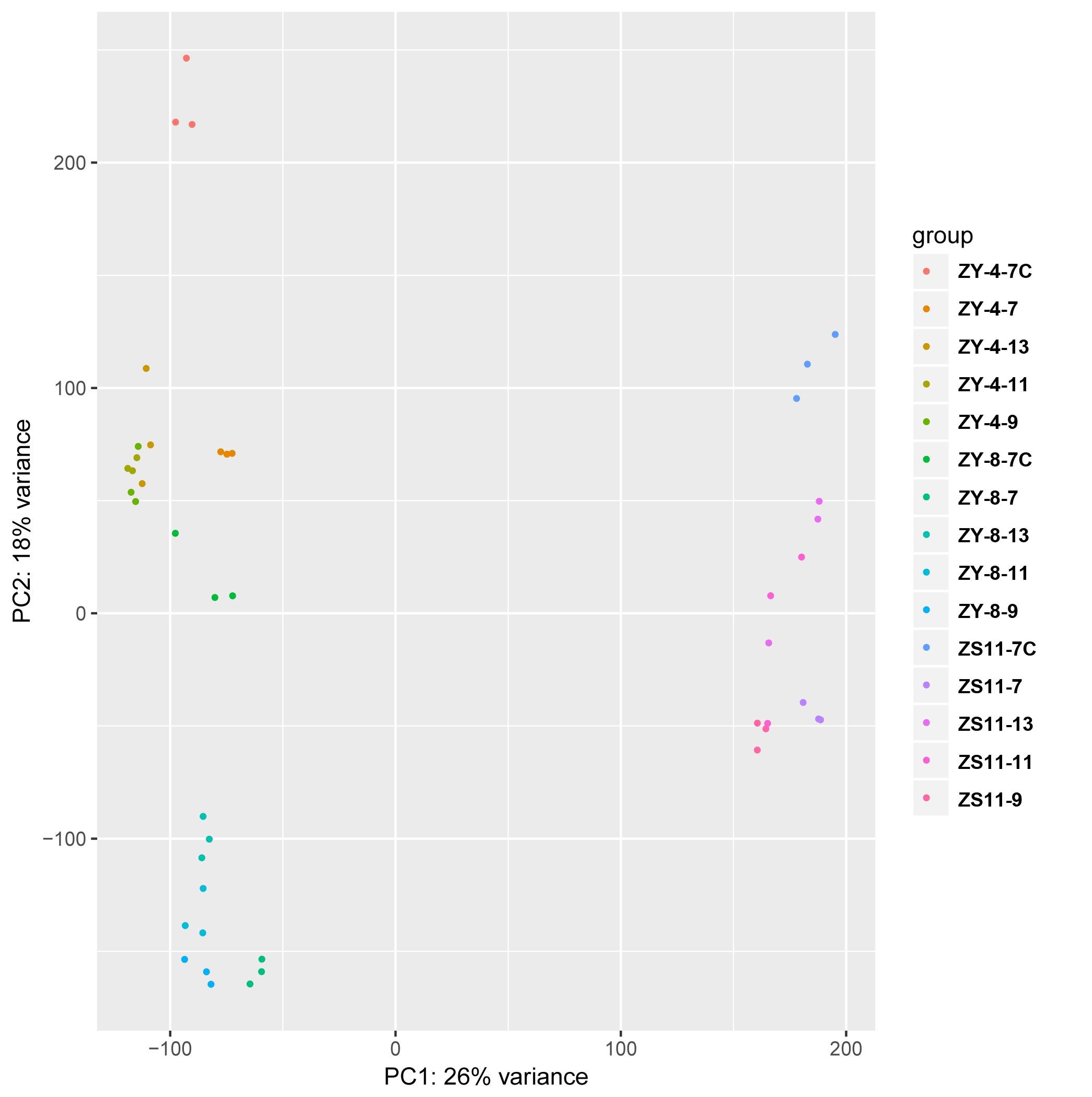

Supplement: Supplementary file 2 — Supplementary file2 (TIF 184 kb) [file 438_2020_1674_MOESM2_ESM.tif]
